# The therapeutic role of self-transcendence in moral injury recovery: *theory, mechanisms, and clinical implications*

**DOI:** 10.3389/fpsyt.2026.1701564

**Published:** 2026-06-10

**Authors:** Wesley H. Fleming

**Affiliations:** Veterans Health Administration, United States Department of Veterans Affairs, Syracuse, NY, United States

**Keywords:** contemplative practice, meditation, mindfulness, moral injury, self-transcendence, spirituality, veterans, meta-awareness

## Abstract

A growing body of psychological and neuroscientific research suggests that moral injury (MI) involves maladaptive self-referential processing, including disruptions in moral identity, rigid negative self-appraisals, and impaired meaning-making following exposure to potentially morally injurious events (PMIEs). Building on Mindfulness-to-Meaning Theory (MMT), this paper proposes self-transcendence (ST)—a metacognitive state characterized by reduced self-focus, expanded awareness, transcendent affect, and prosocial meaning—as a potential mechanism for MI recovery. Within MMT, mindfulness practice is theorized to cultivate ST via decentering and meta-awareness, processes that broaden attentional scope, promote flexible cognitive reappraisal, and modulate habitual self-referential processing. Mindfulness and contemplative research further link ST to increased cognitive flexibility, reduced in shame-focused narrative self-processing, and adaptive integration of emotionally and morally disruptive experiences. Drawing on an integrative review of ST-consistent and MI-related mechanisms, this paper argues that fostering ST through mindfulness-based and contemplative practices may reduce rigid self-focus, expand interpretive frameworks of meaning, and support moral identity repair and meaning-making. Implications are discussed for designing interventions that intentionally cultivate ST as both standalone approaches and modular components, while acknowledging current limitations in measurement, readiness assessment, and the reliable induction of ST states.

## Introduction

“Neither from nor towards; at the still point, there is the dance…” –T.S. Eliot, *Four Quartets* (1).

“The cue to the cure is self-transcendence!” –Viktor Frankl, *Man's Search for Meaning* (2, p. 121).

Professionals working in morally exigent environments—including military personnel, first responders, and healthcare workers—frequently encounter events that violate or profoundly challenge deeply held moral beliefs and ethical commitments, placing them at risk for moral injury (MI) (3–7). MI is increasingly conceptualized as a distinct psychosocial syndrome involving enduring disruptions in moral identity, meaning systems, and self-evaluative processes following exposure to potentially morally injurious events (4, 8–13). Although MI often co-occurs with posttraumatic stress disorder (PTSD), it is widely understood as a distinct moral–psychological condition not fully captured by fear-based trauma models (4, 10, 12, 14). Whereas PTSD centers on threat detection and hyperarousal, MI more centrally involves violations of moral expectations, persistent self-focused moral appraisals, and destabilization of moral identity and meaning (4, 10).

Recent empirical and theoretical literature suggests that MI-related distress is sustained by maladaptive self-referential processing—recurrent rumination, rigid moral self-appraisal, and self-condemning narratives that reorganize identity around perceived transgression or betrayal (8, 10, 15–18). These maladaptive schemas are frequently accompanied by intense moral emotions (e.g., guilt, shame, anger, grief), disruptions of narrative coherence, and existential disorientation, including loss of meaning and relational trust (12, 16, 19, 20). Importantly, MI has also been associated with elevated suicide risk independent of comorbid psychiatric conditions, underscoring its clinical significance (21–23).

Standard evidence-based interventions for posttraumatic stress disorder (PTSD) have shown, across case studies and secondary analyses, only modest symptom reduction among individuals exposed to morally injurious events (24–26). This limited efficacy, together with growing recognition of the conceptual distinctions between MI and PTSD—as reflected in the recent inclusion of MI-related distress under the DSM-5-TR’s Other Conditions That May Be a Focus of Clinical Attention (Z-code; 27)—suggests that MI engages identity-level and existential processes not directly addressed by fear-based treatment models (9, 13, 19). In response, emerging functional and contextual approaches are being developed to more directly target the moral, relational, and meaning-making disruptions central to MI. These interventions aim to restore moral identity, repair relational trust, and facilitate the integration of meaning through processes such as narrative reconstruction, acceptance, values clarification, forgiveness, and grief work (28–32).

Building on this evolving clinical and theoretical landscape, this paper proposes that self-transcendence (ST)—conceived as a cultivable experiential state marked by reduced self-referential processing, broadened metacognitive awareness, self-transcendent affect, and prosocial meaning-making— may represent a promising therapeutic pathway for moral injury (MI) recovery (33–35). Emerging empirical and theoretical work on mindfulness, meaning-making, and self-transcendent experience suggests that ST-related processes are associated with greater psychological flexibility, enhanced emotion regulation, increased connectedness, and more adaptive integration of challenging experiences (34–39). Consistent with these findings, ST is hypothesized to target core processes of MI—such as maladaptive self-referential processing, rigid moral self-appraisals, and disrupted meaning-making (8, 12, 15, 16, 40, 41)—by modulating maladaptive self-referential processing, loosening rigid moral self-appraisals, and facilitating reorganization of meaning and perspective surrounding morally distressing events (33, 34, 42).

To advance this proposition, the paper (1) reviews and analyzes the psychological and neuroscientific foundations of ST to identify a set of ST-consistent mechanisms of change, with particular attention to the Mindfulness-to-Meaning Theory (MMT); (2) examines the functional relevance of these mechanisms to core MI domains; and (3) considers the potential role of ST itself as a transdiagnostic mechanism of change in current and future MI interventions. It should be acknowledged at the outset that direct measurement of ST—particularly as a mediating process in MI recovery—is largely absent from the current literature. The evidence presented here, both empirical and theoretical, is therefore indirect and derived from related intervention domains in which ST is typically inferred rather than explicitly operationalized or tested. Consequently, the causal and mediational claims advanced in this paper should be viewed as theoretically grounded but provisional, reflecting a synthesis of converging, yet indirect, lines of evidence.

## Foundations of moral injury

Moral injury has been conceptualized as arising from events—or cumulative experiences—that violate deeply held moral commitments and disrupt moral self-coherence in relation to oneself, others, and, for some, a higher moral or spiritual framework (4, 12, 13, 16, 20). Clinically, MI is characterized by entrenched moral appraisals (e.g., “I am unforgivable”), identity-level disturbances, and enduring moral emotions such as guilt, shame, anger, grief, and despair. It is also accompanied by downstream sequelae, including social withdrawal, diminished trust, erosion of meaning and purpose, and—in more severe presentations—self-harming behaviors and suicidality (8, 13, 19, 21, 22, 43).

While Posttraumatic stress disorder (PTSD) and moral injury (MI) often co-occur, both are conceptually distinct: PTSD is defined by fear-based responses to life threat (e.g., hyperarousal, re-experiencing, and avoidance of trauma cues), whereas MI centers on transgressions of deeply held moral beliefs and is marked disrupted moral meaning (10, 14, 19). Recent diagnostic developments—including the DSM-5-TR’s Z-code for moral, religious, or spiritual problems (Z65.8)—acknowledge that clinically significant distress may extend beyond traditional PTSD symptom clusters to encompass moral, identity, and existential concerns that warrant explicit assessment and intervention (27).

Potentially morally injurious events (PMIEs) are conventionally categorized as: (1) participating in, or failing to prevent, actions that violate one’s moral code; (2) witnessing extreme violence or moral transgressions; and (3) experiencing betrayal by trusted authorities (4, 44). Within these categories, events that conflict with fundamental moral assumptions—particularly when personal culpability is unclear—pose significant challenges to meaning-making (4, 13, 16, 45–47). Such complex PMIEs often entail moral dilemmas or seemingly senseless tragedies that constrain moral agency, thereby limiting the effectiveness of traditional moral-repair strategies (13, 45, 46).

### Self-referential processing

At the cognitive–affective level, MI-related distress has been proposed to involve maladaptive self-referential processing, including rumination, rigid moral judgments, experiential avoidance, and recursive self-evaluation (15, 16, 48, 49). Quantitative studies in veterans suggest that rumination mediates associations between moral injury and symptoms of depression, anxiety, and PTSD, underscoring the potential role of recursive self-focused processing (15, 49). Parallel civilian research links event-related rumination and reduced cognitive flexibility with heightened shame, providing independent support for this proposed mechanism (48).

Developmental and meaning-making models similarly propose that MI may persist when morally salient experiences are processed through maladaptive, self-referential frameworks that are self-condemning or identity-threatening, thereby disrupting narrative coherence (8, 16, 50). In a related vein, Litz and Walker’s (13) social–functional model proposes that both internalized moral injury—self-directed distress following one’s own perceived transgressions—and externalized, trust-violation moral injury—distress following perceived betrayal by others or institutions—operate through meaning-making processes that shape moral self-evaluations, organize self- and other-schemas, and constrain relational expectations, even though the distress arises from different sources. Acts of betrayal, for example, while originating externally, may be construed as reflections of one’s value, agency, or moral standing within a relational or institutional context (8, 13, 18, 44). An individual might, in such cases, interpret these external violations of trust through self-referential appraisals such as, “I was expendable,” “I was naïve,” or “My trust made me complicit.”

Extending these psychological accounts, neurobiological findings suggest that moral injury is centrally self-focused, involving disruptions in self-related processing. Emerging studies associate moral injury with altered activity in large-scale brain networks supporting self-referential thought, particularly the Default Mode Network (DMN) (40, 41, 51, 52). Investigations of morally salient autobiographical recall report atypical posterior DMN activation, altered connectivity with sensorimotor and midbrain regions, and associations between shame intensity and dorsomedial prefrontal activity—patterns broadly consistent with intensified moral self-focus and difficulties integrating emotionally charged experiences into coherent narratives (40, 41, 52). Resting-state findings likewise suggest heightened self-referential processing in core DMN hubs during betrayal-related moral injury, as evidenced by increased amplitude of low-frequency fluctuations in medial prefrontal and precuneus regions that covary with rumination severity (51). Together, these data indicate that even externally oriented, betrayal-related moral injury may be instantiated as altered self-referential dynamics within core DMN nodes.

Consistent with these views, emerging theories on MI treatment increasingly frame moral injury as a multidimensional phenomenon—encompassing interlocking self-focus, relational, and meaning-making processes—and have informed the development of interventions that prioritize identity-level disruptions and maladaptive meaning-making over symptom reduction alone (13, 53, 54). Current treatment models therefore seek to restore moral and relational functioning, rebuild trust and belonging, cultivate restorative moral and social experiences, and foster acceptance, forgiveness, self-reorientation, and renewed engagement with moral communities (13, 53, 54)—core therapeutic aims that require broadened awareness and reduced self-focus.

Considered together, psychological, relational, and neurobiological findings suggest that therapeutic change may depend on processes that loosen maladaptive self-focus, increase cognitive and moral flexibility, and situate painful experiences within broader relational and existential contexts. These shifts can, in turn, support the integration of morally injurious events into a more coherent narrative identity, restore belonging and trust, and foster a reorganization of the moral self and broader moral worldview. Against this theoretical backdrop, self-transcendent-consistent processes--marked by reduced self-salience and expanded awareness—may emerge as a particularly promising mechanism for transforming maladaptive self-referential processing in moral injury.

## Self-transcendence: operational definition and mechanisms of change

Self-transcendence (ST) is a multidimensional construct spanning philosophy, religion, developmental psychology, and neuroscience. It is broadly defined as a shift in attention, identity, and motivational orientation beyond narrow self-focus toward more expansive and interconnected perspectives (55). Contemporary psychological models increasingly conceptualize ST as a cultivable mode of consciousness characterized by reduced self-salience, attenuation of rigid self–world boundaries, enhanced perceived connectedness, and the emergence of self-transcendent emotions such as compassion and moral elevation (34, 35, 39, 56, 57).

Despite growing interdisciplinary interest, ST remains variably defined and operationalized across empirical and theoretical contexts (55). To enhance conceptual precision and empirical tractability, this analysis integrates these perspectives—drawing especially on Mindfulness-to-Meaning Theory (MMT, 33, 36),—and adopts the following process-based operational definition:

Self-transcendence (ST) is a trainable, multilevel process characterized by coordinated reductions in self-referential processing, expansion of metacognitive awareness, increases in self-transcendent affect, and enhanced prosocial meaning- making, collectively supporting flexible identity and adaptive integration of experience.

This functional definition conceptualizes self-transcendence (ST) as a latent, higher-order construct reflected in four interrelated and measurable domains: (a) reduced self-referential processing, (b) broadened metacognitive awareness, (c) self-transcendent affect, and (d) prosocial meaning-making (see Table1). Within this framework, ST is not reducible to any single domain; rather, it emerges through the dynamic integration of attentional, affective, cognitive, and meaning-oriented processes. This integrative process gives rise to a higher-order configuration—here termed emergent ST—through which individuals develop greater identity flexibility and an enhanced capacity to accommodate morally salient or existentially disruptive experiences. In this sense, emergent ST represents not merely the sum of its constituent domains, but a distinct adaptive mode of self-functioning that facilitates meaning reconstruction, moral integration, and psychological resilience. 

Each domain is amenable to empirical assessment through validated self-report, behavioral, and neurophysiological indices, allowing ST to be examined as a testable, multicomponent mechanism of change. Existing instruments such as the Nondual Awareness Dimensional Assessment, the Mysticism Scale, and indices of self-transcendent emotions provide preliminary tools for capturing both every day and high-intensity manifestations of ST (38, 58). Importantly, ST may manifest both as transient states (e.g., momentary reductions in self-salience) and as trait-like dispositions (e.g., enduring increases in meaning, connectedness, and psychological flexibility).

This process-oriented framing is particularly relevant for moral injury (MI), a condition characterized by rigid, shame-based self-referential processing, moral disintegration, persistent negative affect, and disrupted meaning systems. By targeting the domains outlined above, ST may offer a theoretically coherent pathway for addressing these core features.

### Theoretical framework: mindfulness-to-meaning theory

The present analysis situates ST within the Mindfulness-to-Meaning Theory (MMT; 33, 36), a process model describing how mindfulness-based practices facilitate positive psychological transformation. Within this framework, ST may be understood as an emergent configuration of the four operational domains, arising through recursive interactions among attention, cognition, and affect (see **Table 1**).

**Table 1:** Operational domains and mechanisms of self-transcendence.

ST domain	Brief definition of domain (what changes)	Evidence	Associated mechanisms (how change occurs)	Mechanism description (with APA citations)
(a) Reduced Self-Referential Processing	Decreased engagement in narrative self-focus, evaluative self-referential thought, and rigid self-salience	Reduced DMN activity and rumination linked to mindfulness and ST-related states (59–62)	Decentering; DMN downregulation; attentional disengagement from self-referential loops	Decentering reduces identification with self-related thoughts, weakening habitual narrative processing; associated with decreased DMN activation and reduced rumination (36, 59, 62)
(b) Expanded Metacognitive Awareness	Increased capacity to observe thoughts and emotions as transient events; broadened attentional scope and perspective-taking	Metacognitive awareness mediates mindfulness outcomes and improves cognitive flexibility (36, 63–65)	Attentional broadening; cognitive defusion; perspective-taking; nondual awareness (advanced)	Broadening of attention enables contextual processing and reappraisal; cognitive defusion reduces literal belief in thoughts; advanced states may involve attenuation of subject–object boundaries (36, 38, 66)
(c) Self-Transcendent Affect	Emergence of emotions characterized by reduced self-focus and increased orientation toward others or larger wholes (e.g., compassion, awe, gratitude, moral elevation)	Self-transcendent emotions linked to well-being, emotion regulation, and prosociality (35, 57, 67–69)	Affective broadening; compassion cultivation; awe induction; distress tolerance	Positive, other-oriented emotions broaden the affective field, increase distress tolerance, and counteract avoidance; support integration of negative affect and promote prosocial orientation (61, 67, 69)
(d) Prosocial Meaning-Making	Increased sense of meaning, purpose, and values-based orientation toward others and broader existential frameworks	Meaning in life and values alignment linked to well-being and reduced pathology (35, 70–72)	Value clarification; eudaimonic reappraisal; narrative reconstruction; moral reorientation	Reappraisal processes embed experience within broader moral and existential contexts, supporting identity reconstruction and alignment with prosocial values (33, 71)
Cross-Domain Integration (Emergent ST)	Coordinated, higher-order integration across domains resulting in flexible identity and adaptive meaning	Co-occurrence of attentional, affective, and meaning-related changes in mindfulness and ST research (36, 55, 65, 73)	Upward spirals (MMT); recursive reappraisal; network-level neural integration	Reciprocal interactions among domains generate reinforcing cycles of positive affect and meaning; associated with large-scale network integration (DMN, salience, executive control) (36, 73)
Physiological Regulation (Supporting Process)	Neurophysiological conditions that support integration across domains (not a core domain itself)	Increased HRV and parasympathetic activity linked to mindfulness and regulation (73, 74)	Parasympathetic activation; autonomic regulation; network modulation	Physiological regulation stabilizes attention and affect, enabling sustained engagement and integration across domains (59, 74)

Domains represent latent, measurable dimensions of self-transcendence (ST), whereas mechanisms refer to dynamic processes that produce change within and across these domains. Cross-domain integration reflects the emergent, higher-order organization of ST consistent with Mindfulness-to-Meaning Theory (33, 36).

According to MMT, mindfulness initiates a sequence beginning with decentering from habitual self-referential cognition. Decentering reflects a measurable expansion in metacognitive awareness (domain b), enabling individuals to observe thoughts and emotions as transient mental events rather than identifying with them. This shift simultaneously reduces engagement in self-referential processing (domain a), weakening rigid, evaluative self-narratives that are central to MI.

This decentered stance broadens attentional scope, facilitating positive reappraisal and the savoring of experience (36). As attentional broadening increases, individuals are more likely to access self-transcendent affect (domain c), including compassion, awe, gratitude, and moral elevation (33, 35, 69). These affective states, in turn, may generate insight that reinforces prosocial meaning-making (domain d) by enhancing perceived connectedness, moral concern, and value-based orientation.

Emerging perspectives within contemplative science suggest that decentering may, under certain conditions, deepen into nondual awareness—states characterized by a marked attenuation of subject–object distinctions and self-boundaries (38). Within the present framework, such experiences represent a high-intensity convergence across all four domains, involving profound reductions in self-salience, maximal expansion of awareness, and heightened affective and existential integration.

Across iterative cycles, these processes are proposed to generate “upward spirals” of positive affect and eudaimonic meaning (33, 36, 65). In operational terms, repeated engagement of these domains may consolidate into trait-level shifts, including reduced baseline rumination, increased dispositional mindfulness, enhanced prosocial motivation, and strengthened meaning in life—processes directly relevant to the restoration of moral identity in MI.

## Empirical evidence of therapeutic outcomes

A growing body of empirical research provides convergent—though largely indirect—support for ST as operationalized in the present framework. Across studies, changes in the four domains have been associated with improvements in pain regulation, emotional well-being, prosocial functioning, and addictive behavior (33, 35, 36, 55, 61, 62, 73).

Specifically, reductions in self-referential processing (domain a) have been shown to account for significant variance in pain relief and decreases in maladaptive rumination (61). Increases in metacognitive awareness (domain b) mediate associations between mindfulness and positive health outcomes (64). Elevations in self-transcendent affect (domain c) prospectively predict improvements in mood (57), while shifts in prosocial meaning-making (domain d) are associated with reduced substance misuse and enhanced well-being (72). These domains further covary with underlying neurophysiological processes, such as frontal midline theta activity implicated in self‑regulation, which in turn are linked to improved control over addictive behavior (37).

Importantly, most studies assess these domains independently rather than as a unified construct. Nevertheless, their consistent co-occurrence and reciprocal influence provide convergent support for conceptualizing ST as a latent, multicomponent mechanism. This integrative perspective is particularly relevant for MI, where disruptions span self-processing, affect, and meaning simultaneously. Of course, future empirical work will be needed to test this theoretical account.

## Mechanisms of self-transcendence

Within the operational framework outlined above, mechanisms operating within and across the domains of self-transcendence (ST) are proposed to jointly instantiate ST and contribute to its therapeutic effects on moral injury (MI). In this formulation (see **Table 2**), the domains represent the observable or measurable changes that characterize ST, whereas the underlying mechanisms help explain how these changes emerge and how they may influence core processes implicated in MI through interacting cognitive, affective, behavioral, and neurobiological pathways.

**Table 2:** Mapping self-transcendence mechanisms to moral injury processes.

ST domain (what ST Is)	Primary mechanisms (how ST works)	MI processes targeted	Functional role in MI	Expected clinical outcomes
(a) Reduced Self-Referential Processing	Decentering; attenuation of narrative self-focus; decreased DMN activity; reduced self-salience	Rumination; shame-based self-condemnation; rigid identity narratives	Weakens over-identification with moral failure; reduces evaluative self-focus	Decreased rumination; reduced shame severity; increased psychological flexibility
(b) Expanded Metacognitive Awareness (decentering)	Attentional broadening; cognitive defusion; perspective-taking; nondual awareness (in advanced states)	Experiential avoidance; cognitive rigidity; narrow attentional scope	Enables observation of internal experience without fusion; increases contextual processing	Reduced avoidance; improved emotional tolerance; enhanced reappraisal capacity
(c) Self-Transcendent Affect	Affective broadening; cultivation of compassion, gratitude, awe, moral elevation; distress tolerance	Maladaptive guilt, shame, anger; affective constriction; social disconnection	Transforms and integrates painful moral emotions; increases prosocial emotional states	Improved emotion regulation; increased compassion (self/other); reduced distress
(d) Prosocial Meaning-Making	Value clarification; eudaimonic reappraisal; narrative reconstruction; moral reorientation	Meaning disruption (“worldview collapse”); moral disintegration; impaired forgiveness	Reconstructs moral identity; situates experiences within broader existential frameworks	Increased meaning in life; values-consistent action; enhanced moral coherence
Cross-Domain Integration *(Emergent ST)*	Recursive upward spirals (MMT); integration of attention, affect, and meaning; network-level neural regulation (DMN–salience–executive)	Global MI syndrome (interacting cognitive, affective, existential disruptions)	Coordinates shifts across domains; supports adaptive integration of morally injurious experiences	Holistic recovery: reduced symptoms, restored identity, improved well-being and functioning
Physiological Regulation (Supporting Process)	Parasympathetic activation; increased heart rate variability; large-scale network modulation	Hyperarousal; emotional dysregulation; stress reactivity	Stabilizes neurophysiological conditions for processing and integration	Improved stress regulation; increased resilience; sustained engagement in therapy

The table outlines self−transcendence (ST) domains and mechanisms, along with plausible adaptive links to core processes associated with moral injury (MI). It is intended as a theory−driven conceptual alignment rather than a one−to−one empirically validated mechanism map. The framework is primarily informed by the Mindfulness−to−Meaning Theory, which posits that mindfulness and self−transcendent states promote decentering, broadened awareness, positive reappraisal, and eudaimonic meaning−making as central processes of therapeutic change (36, 61). Citations for specific ST and MI processes are provided in the text.

The sections that follow examine each domain in turn. Each subsection first describes the relevant domain-level transformation associated with ST and then outlines candidate mechanisms theorized to facilitate these changes, along with their potential implications for the alleviation or transformation of MI-related distress.

### Reduced self-referential processing and self-salience (domain a)

At the domain level, reduced self-referential processing involves decreased narrative self-focus and self-salience, including diminished evaluative and self-condemning thought. This configuration reflects a loosening of rigid, shame-based self-narratives common in MI.

Mechanistically, these shifts are linked to downregulation of default mode network (DMN) activity implicated in rumination and autobiographical processing (59, 60, 62). Operational indicators include reduced rumination, less self-concept rigidity, and altered DMN activation. Functionally, such changes may attenuate entrenched self-condemnation and create psychological space for more flexible, context-sensitive engagement with morally salient experiences.

### Expanded metacognitive awareness (domain b)

Decentering reflects an expanded metacognitive awareness and attentional scope (domain b), such that thoughts and emotions are experienced as transient mental events rather than self-defining attributes (63). Mechanistically, decentering and metacognitive monitoring are associated with greater cognitive flexibility, reduced emotional reactivity, and improved contextualization of experience (34, 36, 65). These processes loosen rigid self-identification—often described as nonattachment or flexible self-representation (34, 66). In the context of MI, such mechanisms may support disengagement from absolutistic moral self-judgments, thereby facilitating more adaptive reappraisal and moral reasoning.

### Self-transcendent affect (domain c)

This dimension is defined by the emergence of self-transcendent affect, including compassion, gratitude, awe, and moral elevation—which is characterized by reduced self-focus and increased orientation toward others or broader systems (68, 69). Mechanistically, these emotional states support affective integration by broadening the affective field and increasing tolerance for distressing internal states. Empirical evidence suggests that such emotions enhance emotion regulation, increase distress tolerance, and reduce experiential avoidance (57, 67). Within MI, these processes may counteract affective constriction and moral isolation, fostering compassion toward self and others while preserving moral awareness and accountability.

### Prosocial meaning-making (domain d)

This component reflects shifts toward meaning, purpose, and prosocial engagement, with greater salience of eudaimonic values and a reorientation toward moral coherence and relational connection (35, 70, 71). Processes of value clarification and prosocial orientation support meaning-making by situating experiences within broader moral and existential frameworks. In MI, where moral identity is often fragmented, they may facilitate reconstruction of a values-based identity that can hold both moral failure and ongoing commitment to repair, as reflected in increased sense of meaning, clearer values, and improved alignment between values and behavior.

### Nervous system regulation

At the physiological level, ST-consistent processes are associated with patterns of neurophysiological regulation that both reflect and promote integration across the four functional domains described above (37, 73). In this framework, such patterns are best understood as mechanistic pathways and potential biomarkers of ST-related change, rather than as constituting a distinct domain of ST in their own right. This regulatory mechanism involves modulation of large-scale brain networks—such as the default mode, salience, and executive control networks—together with increased parasympathetic activity, typically indexed by higher heart rate variability (59, 73, 74). These changes may serve as physiological correlates of reduced self-referential processing, enhanced attentional flexibility, and improved affective regulation (59, 73, 74). Within MI, such neural and autonomic regulation may help counter chronic hyperarousal, enabling sustained yet tolerable engagement with morally distressing experiences and fostering more comprehensive integrative processing.

Altogether, these domain-level and physiological processes may, through reciprocal interactions, give rise to emergent configurations of self-transcendence characterized by flexible identity, broadened awareness, and adaptive meaning-making. In terms of the Mindfulness-to-Meaning Theory, such configurations can be understood as “upward spirals” in which attentional broadening, self-transcendent affect, and prosocial reappraisal become dynamically coupled with large-scale neural network integration, providing a higher-order pathway through which ST may restore moral coherence in moral injury.

In sum, self-transcendence, as operationalized here, offers a multicomponent, empirically tractable framework for addressing the core disruptions of moral injury by delineating coordinated changes in shame-based self-processing, affective constriction, and shattered meaning systems. Conceptualizing ST as a latent mechanism within this framework provides a basis for testing whether coordinated shifts across these domains mediate therapeutic gains in moral distress, shame, and functional impairment. At present, however, this account remains provisional, and future research in moral injury populations will need to concurrently assess these domains, incorporate physiological indices of regulation, and evaluate the causal status of ST as a mechanism of change across diverse interventions and moral contexts (**Table 2**).

## Theoretical rationale: how self-transcendence addresses moral injury

Building on the preceding operationalization of self-transcendence (ST), the following theoretical rationale (**Table 3**) illustrates potential therapeutic pathways through which ST may influence core processes implicated in moral injury (MI). Central features of moral injury are examined in relation to ST domains and their associated mechanisms, highlighting how processes linked to ST may contribute to moral injury recovery.

**Table 3:** Mapping moral injury processes to self-transcendence mechanisms.

Moral injury core process	Conceptual definition (MI literature)	Potentially engaged ST-consistent mechanisms (with citations)	Illustrative effects on MI recovery
Experiential avoidance	Efforts to suppress, control, or escape morally painful memories, emotions, and somatic sensations, thereby obstructing moral processing and repair (4, 29, 75).	Decentering and reduced self-referential processing (33, 34, 36, 38).​ Attentional broadening and shifts toward more inclusive frames of meaning (33, 36, 65).​ Expanded awareness that supports non-reactive contact with difficult inner experience (35, 62). ​	ST states may loosen identification with morally painful inner experiences, lowering avoidance and enabling fuller moral and emotional processing.
Rigid moral appraisals/inflexible meaning-making	Over-accommodated, self-condemning moral schemas that constrain reappraisal and moral repair (4, 13, 15, 16, 20).	Broadened attentional scope and perspective (33, 36, 65).​ Enhanced cognitive reappraisal and positive re-framing of adversity (36, 65).​ Orientation toward more expansive meaning structures characteristic of ST experiences (35). ​	ST-related cognitive expansion may foster more nuanced re-interpretations of morally injurious events, supporting flexible meaning-making and moral repair.
Maladaptive self-referential processing (rumination, negative self-focus)	Persistent self-blame, shame, and evaluative self-focus linked to MI severity and dissociative processes (12, 15, 17, 76).	Altered default mode network activity and reduced evaluative self-focus during ST-related states (34, 34, 36). ​ Expanded or allocentric sense of self, including body-boundary dissolution (34, 62). ​ More compassionate, less reified self-relating associated with self-transcendent experiences (64, 72, 77). ​	ST mechanisms may quiet self-critical rumination, reduce self-condemnation, and permit more compassionate self-orientation.
Disruption of core beliefs and meaning (“worldview collapse”)	Shattered assumptions about self, others, and moral order leading to existential disorientation (4, 8, 11, 13, 16).	Reduced self-referential processing with a shift toward more expansive frames of meaning and perspective (35, 62). Decentering, broadened perspective, and positive affect linked to cognitive reappraisal and meaning-making in mindfulness-to-ST models (33, 36, 65).​ Nondual awareness described as “open, empty cognizance” that can support ontological reappraisal (66). ​	ST-related shifts may scaffold reconstruction of disrupted global meaning systems and support existential reintegration during MI recovery.
Grief-related responses to moral loss	Affective and existential grief associated with irreversible moral and spiritual losses, including loss of innocence or moral coherence (8, 13, 47).	Nondual awareness described phenomenologically as wholeness, completeness, or “silent contentment,” without a felt lack (38, 62, 66). Open, boundless awareness in which self–world boundaries relax and experience is felt as intrinsically sufficient (34, 62). Decreased defensive reactivity to painful content within such states (33). ​	ST experiences may allow existential and moral losses to be acknowledged and integrated with greater equanimity, rather than chronically avoided or resisted.
Impaired forgiveness and moral repair	Difficulties in forgiving self or others, impeding moral repair, reconciliation, and relational restoration (8, 12, 16, 20, 78, 79).	Loosening of rigid self-appraisals and evaluative self-processing during ST-related states (34, 36). ​ Broadening identification beyond the individual self, fostering connectedness with others (35, 69). Cultivation of compassion toward self and others in contemplative and ST-related practices (67, 72).	By reducing self-focused evaluative rigidity and enhancing prosocial, compassionate orientation, ST states may create conditions conducive to forgiveness of both self and others.
Persistent negative moral emotions (shame, guilt, anger, disgust)	Sustained self-conscious and moral emotions that maintain MI-related distress and are linked to PTSD, depression, and suicidal ideation (4, 12, 14, 16, 20, 21).	Induction of self-transcendent emotions—such as awe, elevation, and gratitude—defined as emotions that decrease self-salience and orient toward larger realities or others’ welfare (35, 69, 80). Links between self-transcendent emotions and more integrated affective functioning, eudaimonic well-being, and meaning (33, 64, 67, 70, 81). ​ Mindfulness-to-ST models in which cycles of decentering, broadening, and reappraisal transform emotional responses over time (34, 36). ​	ST-related affective processes may help transform, or more cautiously re-contextualize, morally painful emotions within broader, meaning-laden perspectives that support integration rather than chronic dysregulation.

The table outlines core moral injury (MI) processes and plausible adaptive links to mechanisms associated with self-transcendence (ST). It is intended as a theory-guided conceptual alignment rather than a one-to-one empirically validated mechanism map and is primarily informed by the Mindfulness-to-Meaning Theory, which proposes that mindfulness and self-transcendent states foster decentering, broadened awareness, positive reappraisal, and eudaimonic meaning-making as key processes of therapeutic change (33, 36).

### Experiential avoidance

Experiential avoidance—the tendency to suppress or evade morally painful internal experiences—is a central maintaining factor in MI (4, 29, 75). Within the present framework, avoidance reflects dysregulation across multiple ST domains, including rigid self-referential processing (domain a), constricted awareness (domain b), impaired integration of affect (domain c), and heightened nervous system arousal.

Mechanistically, mindfulness initiates decentering--a process within the broadened metacognitive awareness domain—that enables individuals to observe distressing thoughts and emotions without over-identifying with them (36). This shift concurrently reduces engagement in self-referential processing (domain a), weakening the personalization of distress. As these mechanisms unfold, attention broadens and becomes less dominated by threat- or self-focused salience.

Cultivating ST states may deepen this process by further attenuating self-salience and loosening self–world boundaries, thereby enabling distressing moral content to be experienced without defining the self. In this way, coordinated changes across domains (a) and (b) (reduced self-referential processing and broadened metacognitive awareness) supported by emerging affective tolerance (domain c), may reduce avoidance and facilitate adaptive engagement with morally salient material. Concurrently, associated nervous system regulation may mitigate hyperarousal and support the integrative psychological work required for moral repair.

### Rigid moral appraisals and inflexible meaning-making

Moral injury is often sustained by rigid, over-accommodated moral appraisals and self-condemning schemas that constrain meaning-making (4, 15, 16). These patterns reflect inflexibility across reduced self-referential processing and prosocial meaning-making (domains a and d), in which identity-bound interpretations limit the reconstruction of meaning.

ST-related mechanisms, particularly attentional broadening and cognitive reappraisal, operate through expansion of metacognitive awareness (domain b) and reduction of rigid self-referential processing (domain a). As attentional scope widens, individuals gain access to contextual and relational information that supports more nuanced moral evaluations (36, 65).

Simultaneously, activation of prosocial meaning-making (domain d) allows individuals to reinterpret morally injurious experiences within broader existential and relational frameworks. Thus, mechanisms of cognitive flexibility and reappraisal operate across self-referential processing, broadened metacognitive awareness, and prosocial meaning-making (domains a, b, and d), enabling reconstruction of moral meaning and restoring coherence between experience and global belief systems.

### Maladaptive self-referential processing (rumination and negative self-focus)

Heightened rumination and negative self-appraisal are core features of MI (12, 15, 17). Within the ST framework, these processes reflect dysregulation within domain (a), often reinforced by limited metacognitive awareness (domain b).

Mechanistically, decentering disrupts maladaptive self-referential loops by decoupling attention from narrative self-processing (36). Converging neuroscientific evidence suggests that such shifts are associated with altered activity in the default mode network (DMN), corresponding to reduced evaluative self-focus (59, 82).

At higher intensities, ST-related mechanisms may involve attenuation of subject–object boundaries, reflecting convergence across (reduced self-referential processing and broadened metacognitive awareness (domains a and b). These shifts weaken rigid identity narratives and create conditions for more flexible and compassionate self-relating, particularly when accompanied by self-transcendent affect (domain c). Through this coordinated process, rumination is reduced and self-representation becomes less rigid and punitive.

### Disruption of core beliefs and meaning (“worldview collapse”)

Moral injury often involves disruption of global meaning systems, including beliefs about justice, trust, and moral order (11, 16). Within the present framework, such disruptions reflect fragmentation across the prosocial meaning-making domain (domain d), supported by rigid self-referential processing (domain a).

MMT provides a model for how recursive reappraisal processes, initiated through metacognitive awareness (domain b), enable the reconstruction of meaning (33). ST experiences—characterized by reduced self-salience (domain a), expanded awareness (domain b), and increased perceived connectedness (domains c and d)—may further support this reintegration.

Mechanistically, these processes allow individuals to situate morally injurious experiences within broader existential frameworks, facilitating reorganization of disrupted belief systems. The convergence of domains (a–d) supports the transformation of fragmented worldviews into more coherent and inclusive narratives.

### Grief-related responses to moral loss

Moral injury frequently involves grief related to the loss of a morally coherent identity or worldview (83, 84). This reflects disruptions across domains (c) and (d), particularly in the integration of affect and meaning.

ST-related mechanisms, including affective integration and attentional broadening, enable individuals to experience grief without avoidance or over-identification. Within expanded awareness (domain b) and reduced self-referential processing (domain a), grief can be held as part of a larger experiential field.

Self-transcendent affect (domain c)—especially states characterized by connectedness or wholeness--may further transform the phenomenology of loss. These processes support meaning reconstruction (domain d), allowing grief to be integrated into a broader narrative rather than remaining a source of fragmentation.

### Impaired forgiveness and moral repair

Difficulties with forgiveness represent a major barrier to recovery from MI (8, 20). These difficulties reflect rigidity across domains (a) and (d) (reduced self-referential processing and prosocial meaning-making), often accompanied by deficits in self-transcendent affect (domain c).

Mechanistically, ST fosters prosocial orientation and value reorganization, processes primarily situated within domains (c) and (d). By reducing rigid self-identification (domain a) and expanding perspective (domain b), individuals may become more capable of engaging with the complexity of moral situations.

The emergence of compassion and moral elevation (domain c) facilitates forgiveness by supporting empathy and relational awareness. These processes, in turn, enable moral repair by aligning behavior with reconstructed values (domain d).

### Negative moral emotions

Shame, guilt, anger, and moral disgust are central features of MI and are strongly associated with adverse outcomes (14, 21). Within the ST framework, these emotions are often intensified by rigid self-referential processing (domain a) and limited affective integration (domain c).

ST-related mechanisms, particularly attentional broadening and affective transformation, operate across domains (a), (b), and (c). As self-focus diminishes and awareness expands, individuals become more receptive to contextual and relational aspects of experience.

This shift increases the likelihood of self-transcendent emotions such as compassion, gratitude, and awe (domain c), which broaden the affective field and facilitate integration of painful emotions. In conjunction with prosocial meaning-making (domain d), these processes allow negative moral emotions to be recontextualized within broader systems of meaning, supporting transformation rather than suppression (**Table 3**).

## Empirical support for the mediating role of self-transcendence in treatment

Although self-transcendence (ST) has yet to be measured directly in moral injury (MI) intervention studies, several prominent MI treatments appear to engage ST-consistent mechanisms, suggesting that ST may function as a latent or implicit mechanism of change. The following section examines these interventions for conceptual and process-level indications of such mechanisms and their potential contributions to therapeutic change. **Table 4** illustrates the overlap between ST-consistent mechanisms and MI treatments.

**Table 4:** Overlap of self-transcendence mechanisms with moral injury treatment processes.

ST-consistent mechanisms	Mindfulness to Manage Moral Injury MMMI	Acceptance & Commitment Therapy for Moral Injury ACT-MI	Moral repair therapies: Adaptive Disclosure AD/Impact of Killing IOK/Building Spiritual Strength BSS
Reduced self-referential processing & self-salience	Cultivates present-moment, open, nonjudgmental awareness that shifts processing from narrow self-focus to more contextualized perspectives, conceptually aligning with reduced self-referential processing and attenuated self-salience.	Uses self-as-context and cognitive defusion to weaken rigid, shame-based, self-critical cognitions, implicitly attenuating self-focus and loosening identification with self-evaluative content.	AD and IOK challenge global, shame-based self-appraisals by situating morally painful events in broader relational and contextual frames; BSS re-situates experiences within spiritual or transcendent frameworks, reducing rigid, global self-condemnation.
Decentering & metacognitive awareness	Explicitly trains meta-awareness and decentering (observer stance toward thoughts, emotions, sensations), with evidence that increases in decentering during mindfulness practice prospectively predict greater ST experiences.​	Defusion and self-as-context processes are closely analogous to decentering, helping individuals see thoughts and moral pain as events in consciousness rather than defining features of the self.	AD’s imaginal dialogues and structured perspective-taking, IOK’s narrative processing of killing-related memories, and BSS’s relational imagery and spiritual dialogue all foster an observer perspective on morally painful experiences and associated self-judgments. ​
Affective integration & emotion regulation (incl. compassion, reduced self-condemnation)	Encourages nonjudgmental awareness and acceptance of painful moral emotions, alternating mindfulness practice with MI-focused discussion to help participants contact distress without avoidance, increasing compassion toward self and others and reducing blame and condemnation.	Promotes mindful awareness of distress, experiential acceptance, and willingness to contact moral pain while pursuing valued actions; these processes are linked to more compassionate self-perspectives and reduced rigid, shame-based cognitions.	AD and IOK emphasize imaginal exposure, emotional processing, and coherent narrative construction that soften global self-condemnation and support self-forgiveness and self-compassion; BSS reduces spiritual distress and enhances forgiveness via group-based spiritual dialogue and relational exercises.
Broadening of awareness & perspective; non-attachment & flexible self-structure	MBSR-informed training broadens attentional field from narrow self-focus to more contextualized perspectives and supports more frequent positive reappraisal of stressful moral events, loosening rigid self-images.	ACT-MI processes (self-as-context, defusion, psychological flexibility) expand perspective beyond fused self-stories of moral failure and promote non-attachment to rigid self-labels while sustaining contact with moral pain.	AD uses structured perspective-taking with compassionate or morally authoritative figures to expand moral and relational perspective; IOK promotes expanded moral perspective and recontextualization of killing-related experiences; BSS uses group-mediated perspective-taking and psychospiritual development to widen moral and relational frames.
Value clarification & prosocial orientation	MMMI is grounded in a mindfulness-to-meaning model where broadened awareness and positive reappraisal support orientation toward meaning, virtue, and altruism, aiding moral repair and restoration of authentic moral identity.	ACT-MI explicitly targets values-based action and valued living, fostering motivational shifts toward eudaimonic, prosocial, and morally coherent values beyond narrow self-interest.	AD, IOK, and BSS all incorporate meaning-making, values realignment, and amends or action planning that support moral repair, reconnection with moral communities, and prosocial or spiritually coherent engagement.
Nervous system regulation (neural/autonomic)	Mindfulness practices in MMMI (breath, body awareness, acceptance) are modeled on MBSR-like protocols that are associated in the broader literature with parasympathetic activation and modulation of large-scale brain networks supporting emotional stability and integrative awareness.	ACT-MI does not directly target physiology, but mindful awareness and acceptance components parallel processes that, in broader ACT and mindfulness research, are associated with improved autonomic regulation and reduced stress reactivity.	AD, IOK, and BSS do not explicitly present neurophysiological targets, but their emphasis on regulated emotional processing within safe relational and/or spiritual contexts is conceptually compatible with improved autonomic balance and reduced hyperarousal during engagement with morally painful material.

The proposed mechanisms of self−transcendence (ST) and their alignment with MI treatments were derived from an integrative synthesis of prior theoretical and empirical research (e.g., 33–36, 38, 39, 56, 57, 65; and key MI treatment trials cited in the text).

### Mindfulness-based interventions

Mindfulness to Manage Moral Injury (MMMI; 30, 85) is a promising mindfulness-based intervention, with preliminary feasibility data indicating reductions in guilt, shame, and MI related distress among individuals exposed to potentially morally injurious experiences. At a process level, MMMI draws on a Mindfulness Based Stress Reduction (MBSR)–informed model that cultivates present moment, open, nonjudgmental awareness of internal experience (86). This mode of awareness aligns with mechanisms associated with self-transcendence, including reduced narrative self-referential processing (42), enhanced meta-awareness and decentering (34, 38), broadened attentional scope beyond narrow self-focus (33), and increased positive reappraisal of stressors (36). Collectively, these processes reflect a shift from rigid, self-immersed evaluation toward more flexible and contextualized modes of self-experience.

Empirical evidence further indicates that increases in decentering prospectively predict greater self-transcendent experiences, with decentering mediating the effect of mindfulness training on self-transcendent states (34, 39). Together with MMMI’s emphasis on cultivating mindful, decentered awareness, these findings suggest a plausible decentering-to-ST pathway. Although this mechanism has not yet been directly tested in the context of moral injury, it is conceivable that a similar process operates within MMMI.

### Acceptance and commitment therapy for moral injury

Acceptance and Commitment Therapy for Moral Injury (ACT-MI) adapts core ACT processes—mindful awareness of distress, experiential acceptance, self-as-context, and values-based action—to increase psychological flexibility and support perspective taking and meaning making in response to morally injurious experiences (28, 29, 75). Preliminary studies, including a randomized pilot trial with veterans, indicate that ACT-MI is feasible, acceptable, and associated with improvements in psychosocial functioning and valued living (28, 29, 75). Broader ACT research shows that defusion, acceptance, self-as-context, and committed action reduce cognitive fusion and experiential avoidance while enhancing psychological flexibility across clinical populations (87, 88). These processes predict subsequent improvements in mental health, with defusion—conceptually analogous to decentering in the mindfulness literature—and self-as-context showing particularly strong links to reductions in rigid, self-critical, and shame-based thinking, as well as to the development of more compassionate self-perspectives (89).

Although neither ACT nor ACT-MI explicitly targets self-transcendence as a distinct construct, ST processes conceptually overlap with ACT mechanisms in several compatible ways. ST has been described as involving attenuated self-focus, broadened perspective, and value-oriented engagement that extends beyond narrow self-interest (34, 35, 42, 64). These processes closely align with ACT principles that promote flexible self-perspectives (e.g., self-as-context and cognitive defusion), open and accepting engagement with pain and distress, and sustained, values-based action (87, 88). Collectively, this conceptual convergence supports the hypothesis that ACT-MI mechanisms may implicitly foster attenuated self-salience, a broader perspective, and moral reorientation—processes consistent with ST—through which ACT-MI may address moral injury.

### Moral repair–based therapies

Adaptive Disclosure (AD), a combat-specific psychotherapy originally developed for guilt and shame-based posttraumatic stress disorder (PTSD) in service members and later adapted to target moral injury (MI), employs imaginal exposure and relational processing to facilitate moral repair (31). Core procedures include guided dialogues with imagined compassionate or morally authoritative figures, structured perspective-taking, and integration of fragmented memories into more coherent, compassionate narratives (31). Clinically, these methods were designed, in part, to expand moral and relational perspectives and to challenge shame-based self-appraisals by situating morally painful experiences within broader, more compassionate relational and contextual frameworks, thereby engaging processes consistent with self-transcendence (90). Open and controlled trials of AD and AD-enhanced protocols report improvements in PTSD symptoms, depression, and functioning consistent with these proposed processes, although formal mediation analyses remain limited (90, 91).

Impact of Killing (IOK), a brief cognitive-behavioral intervention for MI related to combat killing, promotes structured moral reflection, identification and restructuring of maladaptive killing-related cognitions, narrative emotional processing, cultivation of self-forgiveness and self-compassion, and therapist-supported meaning-making and amends planning within a compassionate therapeutic alliance (22, 32). Trials report reductions in guilt, shame, PTSD symptoms, and functional impairment, consistent with expanded moral perspective, correction of global self-condemnation, and recontextualization of morally painful experiences, though mechanisms have not yet been formally tested in mediation analyses (32, 92). By resituating personal moral pain within a shared human and moral landscape, IOK facilitates moral perspective-taking and compassionate self-understanding, reflecting the identity reorientation characteristic of self-transcendent states.

Building Spiritual Strength (BSS), a group-based intervention targeting MI-related spiritual distress, incorporates spiritual dialogue, empty-chair exercises with a higher power or moral community, relational imagery, and values clarification (93). These processes expand moral and relational perspective through group-mediated perspective-taking and psychospiritual development, while resituating experiences within transcendent or spiritual frameworks via meaning-making and reconnection with more compassionate representations of God. Evaluations demonstrate reductions in spiritual distress and improvements in wellbeing and forgiveness, though causal mediation remains unconfirmed (94). Through vulnerable sharing and reconnection with transcendent sources of meaning, BSS appears to engage ST–consistent processes by broadening moral awareness, fostering a sense of unity beyond the self, softening rigid self-focus, and cultivating self-compassion.

Taken together, findings from mindfulness-based, acceptance-based, and moral repair–oriented treatments for MI suggest the presence of overlapping therapeutic processes that are conceptually consistent with self-transcendence and may operate implicitly. By promoting distance from rigid moral self-appraisals, fostering compassion, broadening perspective, and opening paths to meaning, ST may offer a theoretically tenable unifying mechanism through which diverse interventions alleviate the cognitive, affective, and existential burdens of MI. Direct measurement of self-transcendence in future MI trials will be essential to clarify its role as a mediator and to inform targeted clinical innovation.

## Inducing self-transcendence: empirical foundations and clinical considerations

A growing body of research indicates that self-transcendence (ST) can be cultivated through structured interventions, including mindfulness training, awe-based practices, and psychedelic-assisted therapies (55). When implemented with appropriate methodological and ethical safeguards (73, 95, 96), these approaches are associated with improvements in psychological well-being, adaptive reappraisal, and meaning reconstruction (36, 57, 65, 97–99).

Mindfulness training represents one of the most extensively studied pathways to ST. In a randomized study, Hanley, Dorjee, and Garland (39) found that five brief mindfulness sessions significantly increased both decentering and self-transcendent experience relative to an active control condition, suggesting that even short-term practice can produce measurable ST-related shifts. Core practices such as body scans and mindful breathing have been associated with reductions in self-referential processing and, in some individuals, with diminished perceived body boundaries and shifts toward more allocentric or world-centered framing—features characteristic of ST (34, 35). These findings support the view that ST may emerge as a natural extension of sustained attentional training and decentering. At the same time, outcomes vary as a function of dispositional traits (e.g., absorption, openness), psychological readiness, and contextual factors including program structure, instructor competence, and ethical framing (35, 73, 95, 100, 101). Such variability underscores the importance of individualized and developmentally sensitive implementation.

Awe-based interventions demonstrate comparable potential. Exposure to vast or perceptually expansive stimuli—such as panoramic landscapes, immersive media, or spiritual ritual—can evoke the “small self,” enhance prosocial orientation, and shift existential perspective (68, 98, 102). The “overview effect” reported by astronauts, characterized by profound unity, value reorientation, and diminished self-focus, provides a vivid example of awe-induced ST (103). Although such experiences are context-sensitive and not uniformly replicable, experimental and field studies suggest that scaled or simulated awe stimuli can reliably elicit measurable shifts in self-perception and meaning (104, 105).

Psychedelic-assisted therapies currently offer one of the most robust experimental models for eliciting ST, frequently occasioning ego-dissolution and mystical-type experiences that are associated with sustained reductions in depression, anxiety, and addictive behaviors (97, 106). Therapeutic outcomes appear strongly influenced by psychological “set” (mindset, expectations, preparation), environmental “setting,” and structured post-session integration (96, 99, 107, 108), highlighting the central role of context, support, and integration in optimizing benefit.

Methodological challenges nevertheless remain. Much of the ST literature relies on self-report instruments such as the Self-Transcendence Scale (109) and the Nondual Awareness Dimensional Assessment (38), which—like other contemplative measures—face issues of construct overlap and response bias (110). Moreover, debate persists regarding whether ST is best conceptualized as an emotional state, a shift in self-processing, an existential realization, or a multidimensional construct (55). Continued refinement of measurement strategies and theoretical models will be essential to advance the field.

In sum, current evidence suggests that ST can be elicited across multiple modalities when interventions are thoughtfully structured, titrated to individual readiness, and embedded within supportive relational contexts. For the treatment of moral injury, phase-based and scaffolded models that integrate assessment, psychoeducation, gradual induction, and structured integration may maximize therapeutic potential while preserving safety and tolerability (36, 39, 55).

## Role of chaplains

A substantial body of research supports the involvement of professional health care chaplains in treating moral injury (MI), given their training in moral theory, religious traditions, and spiritual practices (3, 111–113). Chaplain–psychologist co-facilitated groups have been identified as a particularly helpful clinical format in qualitative work examining core components of MI groups (114). A distinctive contribution of chaplaincy is the practice of “listening presence,” a relational and contemplative stance of deep, empathic attentiveness. Parameshwaran (113), drawing on neurophysiological principles and qualitative findings, describes this approach as a “transpersonal/transcendental form of mindfulness” that shifts focus away from the self, fosters metacognitive awareness and self-empathy, and disrupts negative automatic thoughts.

Chaplains’ knowledge of religious traditions and their expertise in contemplative practices—such as prayer, mantra meditation, and yoga (112) —uniquely position them to foster ST. ST appears across faith and wisdom traditions: in Christianity, Judaism, and Islam through prayer and surrender to divine will; in Buddhism through ego-decentering meditation; and in Indigenous traditions through deep connection with nature or the cosmos (115). Drawing on these diverse foundations, chaplains can flexibly adapt practices to individuals’ religious, spiritual, or secular worldviews, thereby enhancing receptivity and therapeutic impact.

## Discussion

The present paper advances the hypothesis that self-transcendence (ST) represents a promising yet underrecognized mechanism for addressing the existential and identity-disruptive dimensions of moral injury (MI). ST is conceptualized as a unifying psychological process, reflected in coordinated changes in self-processing, attention, affect, and meaning, that engages core and proximal MI mechanisms—including experiential avoidance, rigid moral appraisals, maladaptive self-referential processing, and negative moral emotions—while potentially supporting downstream moral–existential repair processes such as meaning disruption, grief related to moral loss, and impairments in forgiveness and reconciliation (4, 13, 36, 65, 71). Although systematic empirical validation remains necessary, this project delineates ST-consistent processes that may function across diverse moral injury interventions, offering a conceptual foundation for future mechanism-driven treatment development.

Therapeutic processes associated with ST are supported across multiple empirical literatures. Mindfulness-based decentering has been linked to greater acceptance, reduced avoidance-oriented responding, and less rigid self-focused appraisal (34, 36, 63). Embodied present-moment practices (e.g., body scan, breath-focused attention, mindful yoga) have been associated with reductions in narrative self-focus (42, 116). Awe- and compassion-inducing experiences are related to diminished self-salience and social isolation (34, 68, 69), whereas values- and meaning-oriented interventions support existential reorientation and purpose reconstruction (36, 65, 71). Collectively, these data underscore the potential of ST as a mechanism of change in psychological processes implicated in MI, including affective reactivity, rumination, inflexible self-schemas, and constricted perspective.

### Self-transcendence as a latent process in existing MI interventions

Processes consistent with ST may already operate implicitly within several MI interventions, even when ST is not explicitly theorized or measured as a mechanism of change. Emerging MI treatments frequently incorporate shifts in self-processing characterized by reduced narrative self-focus, increased decentering, broadened attentional scope, affective integration, and enhanced orientation toward meaning and connection beyond the individual self (29–32, 94). Mindfulness to Manage Moral Injury (MMMI), for example, cultivates metacognitive awareness and attentional broadening—processes empirically associated with decentering and meaning-oriented cognitive–affective reappraisal (30, 36, 39, 85). Similarly, Acceptance and Commitment Therapy for Moral Injury (ACT-MI) employs self-as-context and values clarification to foster flexible perspective-taking and values-guided, meaningful action (28, 29, 75). These mechanisms parallel ST-consistent processes such as mindful affective integration and eudaimonic meaning-making. Moral repair–oriented interventions like Adaptive Disclosure use imaginal dialogue and perspective-taking to promote movement from rigid, self-condemning narratives toward more expansive, contextualized accounts (31, 90), which conceptually overlap with ST-related perspective shifting and mindful positive reappraisal (33, 36, 65).

Across these approaches, improvements in psychological flexibility, reductions in self-focused rumination, gains in psychosocial functioning, and decreases in MI-related distress or moral pain (28, 30, 75, 90, 117) may partly reflect the operation of ST-consistent processes. Although ST has not yet been directly examined as a mediating mechanism, converging evidence of overlapping processes—including decentering, broadened awareness, affective integration, eudaimonic meaning-making, and reappraisal—suggests that ST may function as a latent therapeutic pathway underlying observed clinical gains (36, 39).

### Clinical and research implications

Conceptualizing self-transcendence (ST) as a mechanism of change in moral injury (MI) treatment has two complementary clinical implications: ST may be (a) strategically embedded within existing evidence-based interventions, and/or (b) developed as the primary target of a standalone, process-focused treatment. Each pathway carries distinct advantages, clinical considerations, and implementation requirements.

### Embedded ST within existing MI treatments

Embedding ST within established interventions—such as mindfulness-based approaches (e.g., MMMI), acceptance-based treatments (e.g., ACT-MI), and narrative or moral repair–focused therapies—offers a pragmatically feasible and clinically conservative pathway. In this model, ST is not introduced as a distinct or explicit target, but rather as a latent process intentionally cultivated through existing therapeutic components, including decentering, values clarification, compassion practices, and meaning-making interventions.

To strengthen clinical implementation, structured guidance for screening, titration, and integration is essential. Clinicians should begin with readiness assessment, evaluating dissociation, psychotic vulnerability, trauma severity, and affect regulation capacity using validated measures (e.g., PCL-5, LEC-5) alongside MI-specific instruments (MIOS; 12). Clients demonstrating significant instability may require extended stabilization prior to engaging ST-relevant practices.

Titration within embedded approaches should follow a graded progression. Early phases emphasize attentional control and metacognitive awareness (e.g., brief mindfulness practices), which indirectly reduce rigid self-referential processing. As treatment progresses, clinicians may introduce ST-adjacent practices—such as compassion training, gratitude exercises, or perspective shifting—while maintaining flexibility and monitoring for dysregulation. In this context, ST emerges gradually as a byproduct of cumulative therapeutic processes, rather than as an explicit intervention target.

Integration remains a central mechanism of change. Clinicians should explicitly link shifts in awareness (e.g., reduced self-condemnation, increased connectedness) to moral meaning, identity reconstruction, and values-consistent action. Narrative processing, reflective dialogue, and values-based behavioral commitments help ensure that ST-consistent experiences contribute to moral repair rather than avoidance (29, 118).

This embedded model may be particularly appropriate for high-risk or clinically complex populations, as it allows for incremental exposure to ST processes within familiar therapeutic frameworks, thereby minimizing destabilization while enhancing depth of treatment.

### Standalone ST intervention

In contrast to integrative approaches, a standalone self-transcendence (ST) intervention positions ST as the primary therapeutic target, operationalized within a structured, phase-based protocol. This model enables the deliberate and systematic cultivation of core ST processes, including attenuation of maladaptive self-referential processing, expansion of relational and contextual awareness, and reorganization of moral identity.

Given its intensity and focus, a standalone ST intervention requires heightened clinical specificity and a robust safety infrastructure. Comprehensive screening procedures should assess not only the severity of moral injury (MI) and trauma exposure, but also vulnerability to dissociation, psychosis risk, and readiness for contemplative practices. Recommended instruments include the Dissociative Experiences Scale–II (DES-II; 119), the PRIME Screen–Revised for psychosis-risk symptoms (120), and the Mindful Attention Awareness Scale (MAAS; 121). Exclusion criteria or protocol modifications may be warranted in cases of active psychosis, severe dissociative symptomatology, or limited capacity to sustain present-centered awareness.

Careful titration is especially critical within this model. Treatment should proceed through a progressive, scaffolded sequence, beginning with stabilization and attentional training, and gradually advancing toward more explicitly self-transcendent practices (e.g., compassion meditation, awe induction, and nondual awareness practices). Clinicians should continuously monitor arousal levels, dissociative symptoms, and integrative capacity, adjusting intervention intensity accordingly. Importantly, ST practices should be explicitly framed as temporary, flexible shifts in perspective that support adaptive engagement with moral pain, rather than as forms of avoidance or transcendence of moral responsibility.

Integration processes are indispensable to the effectiveness of a standalone ST intervention. Each session should include structured debriefing aimed at translating transient ST experiences into enduring changes in moral cognition, identity, and relational functioning. Evidence-informed techniques such as narrative reconstruction, guided reflection, and values clarification are essential for consolidating state-level experiences into trait-level transformation.

Clinicians should provide brief psychoeducation regarding integration (e.g., conceptualizing insights as “seeds” requiring ongoing cultivation) and normalize variability in the emergence of meaning over time. Structured reflection prompts may facilitate consolidation of ST-related insights, such as: “What felt different about your sense of self when the inner critic softened?”; “What became possible in that space?”; and “How does connect to what matters most in your relationships or moral commitments?”

To further support integration, clinicians may incorporate 15–20 minute expressive writing or narrative exercises designed to revise rigid or self-condemning narratives (e.g., shifting from “unforgivable” to “deeply flawed yet committed to repair”) and to promote future-oriented reauthoring (e.g., “What title would you give the next chapter of your life?”). Experiential techniques such as imaginal dialogue (e.g., empty-chair work or dialogue with a younger self) may also be employed to facilitate self-forgiveness, moral accountability, and reparative intention.

Sessions should conclude with collaborative action planning that translates emergent meaning into concrete, values-consistent behavioral steps (e.g., identifying one action in the coming week that reflects moral repair or relational commitment). Between-session practices, including daily or near-daily journaling, may be assigned to track alignment among values, actions, and evolving self-narratives (e.g., “Today I lived into my values by…”). Clinicians should explicitly acknowledge ambivalence and fluctuations in motivation as normative, and should revisit emergent narrative themes longitudinally to support increasing coherence, flexibility, and integration over time.

In terms of implementation, standalone ST interventions may be delivered in individual or group formats. Individual delivery allows for precise titration and risk management, whereas group contexts may enhance shared meaning-making, normalization, and collective forms of transcendence, particularly in veteran or morally exposed populations.

Finally, both embedded and standalone approaches require cultural and spiritual responsiveness. ST experiences are often interpreted through existential or religious frameworks; thus, clinicians should adopt a collaborative stance and, when appropriate, integrate chaplaincy or spiritual care.

## Future research

Future research on self-transcendence (ST) in moral injury (MI) should prioritize three aims: (a) refining multilevel measurement, (b) testing ST as a multicomponent mechanism of change within existing MI interventions, and (c) developing and evaluating both embedded and standalone ST-based treatments.

### Measurement refinement

Foundational measurement work is needed to operationalize ST as a higher-order latent construct reflected in coordinated changes across four domains: reduced self-referential processing, broadened metacognitive awareness, self-transcendent affect, and prosocial meaning-making. This requires developing and validating domain-specific subscales and testing their higher-order structure using factor-analytic or network models. To avoid mono-method bias, multimodal assessment approaches should integrate self-report data with behavioral tasks and physiological markers (e.g., heart rate variability, large-scale network dynamics).

### Testing ST as a mechanism of change within existing MI treatment

Longitudinal and experimental research should examine whether ST functions as a multicomponent mechanism of therapeutic change within existing MI interventions. The proposed ST battery could be embedded in randomized trials of mindfulness-based, acceptance-based, spiritually integrated, or meaning-centered therapies. These studies should assess whether coordinated shifts across the four domains mediate reductions in shame, guilt, moral disintegration, and functional impairment. Intensive longitudinal designs (e.g., ecological momentary assessment, session-by-session measures) can help model how micro-level processes—momentary decentering, transient experiences of awe or compassion, and daily shifts in meaning—accumulate into trait-level changes predicting moral repair, forgiveness, and moral identity restoration.

### Novel embedded and standalone interventions

For novel embedded ST interventions, randomized controlled trials (RCTs) of existing MI treatments (e.g., MMMI, ACT-MI, Adaptive Disclosure) should include longitudinal measures of ST to test its role as a mediating variable (4, 15). Multilevel structural equation modeling or cross-lagged panel analyses can clarify whether increases in ST precede and explain reductions in shame, guilt, and identity disruption (36, 65). Recommended measures include the Nondual Awareness Dimensional Assessment (NADA), the Mysticism Scale, and indices of self-transcendent emotions to capture both every day and high-intensity ST states (38, 58).

Standalone interventions that explicitly target ST should begin with pilot and feasibility trials to assess acceptability, safety, and tolerability in MI populations (75). Subsequent RCTs can compare ST-focused programs to treatment-as-usual, established MI treatments, or structurally equivalent control conditions, thereby testing both clinical efficacy and incremental validity over existing approaches (8, 20).

### Isolating active components and mechanisms

Dismantling studies are needed to isolate active components of ST. Interventions that systematically include or exclude specific elements (e.g., compassion practices, awe inductions, explicit nondual awareness training) can clarify their unique and combined contributions to outcomes such as moral repair, forgiveness, and reductions in self-condemnation (33, 82). Dose–response and temporal sequencing studies using intensive longitudinal methods may further delineate how transient state-level ST experiences accumulate into sustained trait-level change, directly testing predictions from the Mindfulness-to-Meaning framework (36, 65).

### Mixed-methods and multimodal approaches.

Given the multidimensional nature of ST, mixed-methods designs are essential. Qualitative interviews can elucidate how individuals interpret transcendence in relation to moral responsibility, forgiveness, and repair, while distinguishing adaptive ST from avoidance or “spiritual bypassing” (47, 84). Multimodal measurement development should integrate self-report, behavioral, physiological (e.g., heart rate variability), and neurocognitive indicators to comprehensively operationalize ST as a higher-order construct reflected in coordinated change across self-processing, metacognition, affect, and prosocial meaning (55, 73).

### Limitations and safeguards

Several limitations apply across both embedded and standalone models. Most notably, the hypothesis that ST functions as a mechanism of change in MI treatment remains largely untested in MI-specific clinical populations, limiting current conclusions regarding causality and generalizability.

Importantly, ST experiences are not uniformly beneficial and may introduce clinical risk. Individuals with high dissociation, psychotic vulnerability, or severe trauma-related dysregulation may experience destabilization when engaging in practices that attenuate self-boundaries. Accordingly, both models should incorporate explicit safeguards, including:

- Structured screening and readiness assessment prior to ST exposure.
- Phase-based titration, progressing from stabilization to more evocative practices.
- Ongoing monitoring of dissociation, arousal, and adverse reactions.
- Immediate stabilization strategies (e.g., grounding, orienting, breath regulation).
- Flexible pacing and individualized adaptation.

In standalone interventions, these safeguards should be formalized within the treatment protocol, whereas in embedded approaches they may be integrated within existing clinical structures.

A further limitation involves the risk of misinterpretation of ST experiences. Without appropriate framing, clients may construe transcendent states as negating moral responsibility or prematurely resolving moral conflict. Clinicians must therefore emphasize that ST is a means of loosening rigid self-condemnation while maintaining moral engagement, accountability, and commitment to repair.

Cultural and spiritual variability also represent important considerations. ST experiences are shaped by interpretive frameworks that vary across individuals and communities. Failure to account for these differences may result in cultural or spiritual dissonance and raise ethical concerns. Collaborative engagement with spiritual care providers may enhance cultural responsiveness, particularly in morally and existentially complex cases.

Finally, the measurement of ST remains an ongoing challenge. Existing instruments may not fully capture its multidimensional and dynamic nature, particularly in clinical populations with MI. This limitation underscores the need for continued refinement of assessment tools and the use of multimethod approaches.

## Conclusion

Grounded in theoretical rationale and supporting empirical evidence, this paper proposes that self-transcendence (ST) directly addresses core features of moral injury (MI), including experiential avoidance, rigid moral appraisals, maladaptive self-referential processing, impaired meaning-making, maladaptive grief responses to moral loss, forgiveness difficulties, and persistent negative moral emotions. Key ST processes—reduced self-referential processing, broadened awareness, affective integration, and value clarification—may be particularly relevant for remediating MI’s psychological and existential disruptions. ST therefore warrants empirical testing both as a mediating mechanism of therapeutic change and as a potential means of enhancing existing evidence-based interventions.

Intentionally cultivating ST through targeted interventions, whether as a standalone approach or as a modular component—presents substantial opportunities for clinical innovation. Challenges remain, however, in operationalizing ST safely and effectively, given its sensitivity to individual differences, developmental readiness, and cultural context. Robust assessment tools and rigorous evaluation across diverse populations and settings are essential next steps.

## Data Availability

The original contributions presented in the study are included in the article/supplementary material. Further inquiries can be directed to the corresponding author.

## References

[1] Eliot TS . Four quartets. New York: Harcourt, Brace and Co (1943).

[2] Frankl VE . Man's search for meaning: gift edition. Boston, Massachusetts: Beacon Press (2014).

[3] Koenig HG Al Zaben F . Moral injury: An increasingly recognized and wide spread syndrome. J Relig. Health. (2021) 60:2989–3011. doi: 10.1007/s10943-021-01328-0 34245433 PMC8270769

[4] Litz BT Stein N Delaney E Lebowitz L Nash WP Silva C . Moral injury and moral repair in war veterans: A preliminary model and intervention strategy. Clin Psychol Rev. (2009) 29:695–706. doi: 10.1016/j.cpr.2009.07.003 19683376

[5] Mantri S Lawson JM Wang Z Koenig HG . Identifying moral injury in healthcare professionals: the moral injury symptom scale-HP. J Relig. Health. (2020) 59:2323–40. doi: 10.1007/s10943-020-01065-w 32681398 PMC7366883

[6] Rosen A Cahill JM Dugdale LS . Moral injury in health care: identification and repair in the COVID-19 era. J Gen Internal Med. (2022) 37:3739–43. doi: 10.1007/s11606-022-07761-5 35970958 PMC9377663

[7] Williamson V Stevelink SAM Greenberg N . Occupational moral injury and mental health: systematic review and meta-analysis. Br J Psychiatry. (2018) 212:339–46. doi: 10.1192/bjp.2018.55 29786495

[8] Currier JM Holland JM Malott J . Moral injury, meaning making, and mental health in returning veterans. J Clin Psychol. (2015) 71:229–40. doi: 10.1002/jclp.22134 25331653

[9] Currier JM Drescher KD Nieuwsma J . Introduction to moral injury. In: Currier JM Drescher KD Nieuwsma J , editors.Addressing moral injury in clinical practice. Washington, DC, US: American Psychological Association (2021). p. 3–18. doi: 10.1037/0000204-001

[10] Barnes HA Hurley RA Taber KH . Moral injury and PTSD: Often co-occurring yet mechanistically different. J Neuropsychiat. Clin Neurosci. (2019) 31:A4–103. doi: 10.1176/appi.neuropsych.19020036 31012825

[11] Jinkerson JD . Defining and assessing moral injury: A syndrome perspective. Traumatology. (2016) 22:122–30. doi: 10.1037/trm0000069 27371692

[12] Litz BT Plouffe RA Nazarov A Murphy D Phelps A Coady A . Defining and assessing the syndrome of moral injury: Initial findings of the Moral Injury Outcome Scale Consortium. Front Psychiatry. (2022) 12:923928. doi: 10.3389/fpsyt.2022.923928 35873252 PMC9297368

[13] Litz BT Walker HE . Moral injury: An overview of conceptual, definitional, assessment, and treatment issues. Annu Rev Clin Psychol. (2025) 21. doi: 10.1146/annurev-clinpsy-081423-022604 39879547

[14] Bryan CJ Bryan AO Roberge E Leifker FR Rozek DC . Moral injury, posttraumatic stress disorder, and suicidal behavior among National Guard personnel. psychol Trauma.: Theory. Res. Prac. Policy. (2018) 10:36–45. doi: 10.1037/tra0000290 28581315

[15] Bravo AJ Kelley ML Mason R Ehlke SJ Vinci C Redman JC . Rumination as a mediator of the associations between moral injury and mental health problems in combat-wounded veterans. Traumatology. (2020) 26:52–60. doi: 10.1037/trm0000198 32863781 PMC7449509

[16] Currier JM Farnsworth JK Drescher KD McDermott RC Sims BM Albright DL . Development and evaluation of the expressions of moral injury scale— military version. Clin Psychol Psychother. (2018) 25:474–88. doi: 10.1002/cpp.2170 29282787

[17] Held P Klassen BJ Hall JM Friese TR Bertsch-Gout MM Zalta A . ‘I knew it was wrong the moment I got the order’: A narrative thematic analysis of moral injury in combat veterans. psychol Trauma.: Theory. Res. Prac. Policy. (2019) 11:395–405. doi: 10.1037/tra0000364 29723032 PMC6215519

[18] Hodgson TJ Carey LB Koenig HG . Moral injury, betrayal and retribution: Australian veterans and the role of chaplains. Journal of Religion and Health. (2022) 61(2):993–1021. doi: 10.1007/s10943-022-01507-7 35175506

[19] Griffin BJ Purcell N Burkman K Litz BT Bryan CJ Schmitz M . Moral injury: An integrative review. J Trauma. Stress. (2019) 32:350–62. doi: 10.1002/jts.22362 30688367

[20] Koenig HG Ames D Youssef NA Oliver JP Volk F Teng EJ . The moral injury symptom scale-military version. J Relig. Health. (2018) 57:249–65. doi: 10.1007/s10943-017-0531-9 29196962

[21] Khan AJ Griffin BJ Maguen S . A review of research on moral injury and suicide risk. Curr Treat Opt. Psychiatry. (2023) 10:259–87. doi: 10.1007/s40501-023-00293-7 30311153

[22] Maguen S Griffin BJ Vogt D Hoffmire CA Blosnich JR Bernhard PA . Moral injury and peri- and post-military suicide attempts among post-9/11 veterans. Psychol Med. (2022) 53:3200–9. doi: 10.1017/S0033291721005274 35034682 PMC10235653

[23] Griffin BJ Maguen S McCue ML Pietrzak RH McLean CP Hamblen JL . Moral injury is independently associated with suicidal ideation and suicide attempt in high-stress, service-oriented occupations. Npj Ment Health Res. (2025) 4(1):32. doi: 10.1038/s44184-025-00151-9 PMC12317004 40751003

[24] Held P Klassen BJ Brennan MB Zalta AK . Using Prolonged Exposure and Cognitive Processing Therapy to treat veterans with moral injury-based PTSD: Two case examples. Cogn Behav Pract. (2018) 25:377–90. doi: 10.1016/j.cbpra.2017.09.003 30147290 PMC6103315

[25] Smith ER Duax JM Rauch SAM . Perceived perpetration during traumatic events: Clinical suggestions from experts in Prolonged Exposure therapy. Cogn Behav Pract. (2012) 20:461–70. doi: 10.1016/j.cbpra.2012.12.002 38826717

[26] Steenkamp MM Litz BT Hoge CW Marmar CR . Psychotherapy for military-related PTSD: A review of randomized clinical trials. J Am Med Assoc (JAMA). (2015) 314:489–500. doi: 10.1001/jama.2015.8370 26241600

[27] American Psychiatric Association . DSM-5-TR® Update: supplement to the diagnostic and statistical manual of mental disorders, fifth edition, text revision (September 2025 text revision). Washington, DC: American Psychiatric Association Publishing (2025).

[28] Borges LM Barnes SM Farnsworth JK Drescher KD Walser RD . Case conceptualizing in acceptance and commitment therapy for moral injury: An active and ongoing approach to understanding and intervening on moral injury. Front Psychiatry. (2022) 13:910414. doi: 10.3389/fpsyt.2022.910414 35845442 PMC9279691

[29] Farnsworth JK Drescher KD Evans W Walser RD . A functional approach to understanding and treating military-related moral injury. J Context. Behav Sci. (2017) 6:391–7. doi: 10.1016/j.jcbs.2017.07.003 38826717

[30] Kelley ML Strowger M Chentsova VO Bravo AJ Gaylord SA Burgin EE . Mindfulness to manage moral injury: Rationale and development of a live online 7-week group intervention for veterans with moral injury. Contemp. Clin Trial. Commun. (2022) 30:101011. doi: 10.1016/j.conctc.2022.101011 36340697 PMC9626875

[31] Litz BT Lebowitz L Gray MJ Nash WP . Adaptive disclosure: A new treatment for military trauma, loss, and moral injury. New York, NY, US: Guilford Press (2017).

[32] Maguen S Burkman K Madden E Dinh J Bosch J Keyser J . Impact of killing in war: A randomized, controlled pilot trial. J Clin Psychol. (2017) 73:997–1012. doi: 10.1002/jclp.22471 28294318

[33] Garland EL Fredrickson BL . Positive psychological states in the arc from mindfulness to self-transcendence: Extensions of the mindfulness-to-meaning theory and applications to addiction and chronic pain treatment. Curr Opin Psychol. (2019) 28:184–91. doi: 10.1016/j.copsyc.2019.01.004 30763873 PMC6626690

[34] Hanley AW Dambrun M Garland EL . Effects of mindfulness meditation on self-transcendent states: Perceived body boundaries and spatial frames of reference. Mindfulness. (2020) 11:1194–203. doi: 10.1007/s12671-020-01330-9 33747250 PMC7968136

[35] Yaden DB Haidt J Hood RW Vago DR Newberg AB . The varieties of self-transcendent experience. Rev Gen Psychol. (2017) 21:143–60. doi: 10.1037/gpr0000102 27371692

[36] Garland EL Farb NA Goldin PR Fredrickson BL . Mindfulness broadens awareness and builds eudaimonic meaning: A process model of mindful positive emotion regulation. psychol Inq. (2015) 26:293–314. doi: 10.1080/1047840X.2015.1064294 27087765 PMC4826727

[37] Garland EL Hanley AW Hudak J Nakamura Y Froeliger B . Mindfulness-induced endogenous theta stimulation occasions self-transcendence and inhibits addictive behavior. Sci Adv. (2022) 8. doi: 10.1126/sciadv.abo44 PMC9555770 36223472

[38] Hanley AW Nakamura Y Garland EL . The Nondual Awareness Dimensional Assessment (NADA): New tools to assess nondual traits and states of consciousness occurring within and beyond the context of meditation. psychol Assess. (2018) 30:1625–39. doi: 10.1037/pas0000615 30058824 PMC6265073

[39] Hanley AW Dorjee D Garland EL . Mindfulness training encourages self-transcendent states via decentering. Psychol Conscious.: Theory. Res. Pract. (2023) 10:431–40. doi: 10.1037/cns0000262 27371692

[40] Lloyd CS Nicholson AA Densmore M Théberge J Neufeld RW Jetly R . Shame on the brain: Neural correlates of moral injury event recall in posttraumatic stress disorder. Depression Anxiety. (2021) 38:596–605. doi: 10.1002/da.23128 33369799

[41] Terpou BA Lloyd CS Densmore M McKinnon MC Théberge J Neufeld RWJ . Moral wounds run deep: exaggerated midbrain functional network connectivity across the default mode network in posttraumatic stress disorder. J Psychiatry Neurosci. (2022) 47:E56–66. doi: 10.1503/jpn.210117 35177485 PMC8865964

[42] Farb NA Segal ZV Mayberg H Bean J McKeon D Fatima Z . Attending to the present: Mindfulness meditation reveals distinct neural modes of self-reference. Soc Cogn Affect Neurosci. (2007) 2:313–22. doi: 10.1093/scan/nsm030 18985137 PMC2566754

[43] Maguen S Nichter B Norman SB Pietrzak RH . Moral injury and substance use disorders among US combat veterans: Results from the 2019–2020 National Health and Resilience in Veterans Study. Psychol Med. (2023) 53:1364–70. doi: 10.1017/S0033291721002919 34409932 PMC10009375

[44] Shay J . Achilles in Vietnam: Combat trauma and the undoing of character. New York, NY: Simon and Schuster (1994).

[45] Fleming WH . Moral injury and the absurd: The suffering of moral paradox. J Relig. Health. (2021) 60:3012–33. doi: 10.1007/s10943-021-01227-4 33725298

[46] Fleming WH . Complex moral injury: shattered moral assumptions. J Relig. Health. (2022) 61:1022–50. doi: 10.1007/s10943-022-01542-4 35274226

[47] Molendijk T . Toward an interdisciplinary conceptualization of moral injury: From unequivocal guilt and anger to moral conflict and disorientation. New Ideas. Psychol. (2018) 51:1–8. doi: 10.1016/j.newideapsych.2018.04.006 38826717

[48] Cenkner DP Usman H Zalta AK . Differential associations of rumination and cognitive flexibility with guilt and shame following potentially morally injurious events. J Affect Disord. (2023) 325:135–40. doi: 10.1016/j.jad.2022.12.165 36621679

[49] Hamrick HC Kelley ML Bravo AJ . Morally injurious events, moral injury, and suicidality among recent-era veterans: The moderating effects of rumination and mindfulness. Mil. Behav Health. (2020) 8:109–20. doi: 10.1080/21635781.2019.1669509 37339054

[50] Corona CD Van Orden KA Wisco BE Pietrzak RH . Meaning in life moderates the association between morally injurious experiences and suicide ideation among US combat veterans: Results from the National Health and Resilience in Veterans Study. psychol Trauma.: Theory. Res. Prac. Policy. (2019) 11:614. doi: 10.1037/tra0000475 31144841 PMC6722007

[51] Fulton TM Guelfo A Elbasheir A McDermott TJ Lee J Ahluwalia V . Resting-state neural signatures of moral injury: associations with rumination. Biol Psychiatr.: Cogn Neurosci Neuroimaging. (2025). doi: 10.1016/j.bpsc.2025.08.001 40825519 PMC12415846

[52] Kearney BE Terpou BA Densmore M Shaw SB Théberge J Jetly R . How the body remembers: Examining the default mode and sensorimotor networks during moral injury autobiographical memory retrieval in PTSD. NeuroImg.: Clin. (2023) 38:103426. doi: 10.1016/j.nicl.2023.103426 37207593 PMC10206209

[53] Evans WR Smigelsky MA Frankfurt SB Antal CJ Yeomans PD Check C . Emerging interventions for moral injury: expanding pathways to moral healing. Curr Treat Opt. Psychiatry. (2023) 10:431–45. doi: 10.1007/s40501-023-00303-8 30311153

[54] Williamson V Kothari R Bonson A Campbell G Greenberg N Murphy D . Moral injury prevention and intervention. Eur J Psychotraumatol. (2025) 16:2567721. doi: 10.1080/20008066.2025.2567721 41118287 PMC12541919

[55] Kitson A Chirico A Gaggioli A Riecke BE . A review on research and evaluation methods for investigating self-transcendence. Front Psychol. (2020) 11:547687. doi: 10.3389/fpsyg.2020.547687 33312147 PMC7701337

[56] Sacchet MD Fava M Garland EL . Modulating self-referential processing through meditation and psychedelics: is scientific investigation of self-transcendence clinically relevant? World Psychiatr.: Off J World Psychiatr Assoc (WPA). (2024) 23:298–9. doi: 10.1002/wps.21214 38727064 PMC11083968

[57] Hanley AW Mai T Garland EL . Self-transcendent states during a modified mindfulness-based stress reduction program predict improvements in mood. Psychol Conscious.: Theory. Res. Pract. (2023) 10:213–24. doi: 10.1037/cns0000347 27371692

[58] Hood RW Ghorbani N Watson PJ Ghramaleki AF Bing MN Davison HK . Dimensions of the mysticism scale: Confirming the three‐factor structure in the United States and Iran. J For. Sci Study. Relig. (2001) 40:691–705. doi: 10.1111/0021-8294.00085 40046247

[59] Brewer JA Worhunsky PD Gray JR Tang YY Weber J Kober H . Meditation experience is associated with differences in default mode network activity and connectivity. Proc Natl Acad Sci. (2011) 108:20254–9. doi: 10.1073/pnas.1112029108 22114193 PMC3250176

[60] Fox KC Dixon ML Nijeboer S Girn M Floman JL Lifshitz M . Functional neuroanatomy of meditation: A review and meta-analysis of 78 functional neuroimaging investigations. Neurosci Biobehav Rev. (2016) 65:208–28. doi: 10.1016/j.neubiorev.2016.03.021 27032724

[61] Garland EL Hanley AW Riquino MR Reese SE Baker AK Salas K . Mindfulness-oriented recovery enhancement reduces opioid misuse risk via analgesic and positive psychological mechanisms: A randomized controlled trial. J Consult. Clin Psychol. (2019) 87:927–40. doi: 10.1037/ccp0000390 31556669 PMC6764586

[62] Josipovic Z . Neural correlates with nondual awareness in meditation. Ann N. Y. Acad Sci. (2014) 1307:9–18. doi: 10.1111/nyas.12261 24033505

[63] Bernstein A Hadash Y Lichtash Y Tanay G Shepherd K Fresco DM . Decentering and related constructs: A critical review and metacognitive processes model. Perspect psychol Sci. (2015) 10:599–617. doi: 10.1177/1745691615594577 26385999 PMC5103165

[64] Dambrun M Hanley AW Garland EL De Oliveira P Stinus C Pellerin N . The effect of a short mindfulness meditation practice on positive mental health: Self-transcendence as a mediating process. Int J Well-being. (2024) 14. doi: 10.5502/ijw.v14i3.3635

[65] Garland EL Hanley AW Goldin PR Gross JJ . Testing the mindfulness-to-meaning theory: Evidence for mindful positive emotion regulation from a reanalysis of longitudinal data. PloS One. (2017) 12:e0187727. doi: 10.1371/journal.pone.0187727 29211754 PMC5718463

[66] Josipovic Z Miskovic V . Nondual awareness and minimal phenomenal experience. Front Psychol. (2020) 11:2087. doi: 10.3389/fpsyg.2020.02087 32973628 PMC7473343

[67] Fredrickson BL . Positive emotions broaden and build. In: Advances in experimental social psychology, vol. 47. Burlington, MA: Academic Press (2013). p. 1–53. doi: 10.1016/B978-0-12-407236-7.00001-2

[68] Piff PK Dietze P Feinberg M Stancato DM Keltner D . Awe, the small self, and prosocial behavior. In: Journal of personality and social psychology, vol. 108. (2015). p. 883. doi: 10.1037/pspi0000018 25984788

[69] Stellar JE Gordon AM Piff PK Cordaro D Anderson CL Bai Y . Self-transcendent emotions and their social functions: Compassion, gratitude, and awe bind us to others through prosociality. Emotion Rev. (2017) 9:200–7. doi: 10.1177/1754073916684557

[70] Huta V Ryan RM . Pursuing pleasure or virtue: The differential and overlapping well-being benefits of hedonic and eudaimonic motives. J Happin. Stud. (2010) 11:735–62. doi: 10.1007/s10902-009-9171-4 30311153

[71] Wong PTP . Integrative meaning therapy: from logotherapy to existential positive interventions. In: Russo-Netzer P Schulenberg SE Batthyany A , editors.Clinical perspectives on meaning: positive and existential psychotherapy. Cham, Switzerland: Springer International Publishing/Springer Nature (2016). p. 323–42. doi: 10.1007/978-3-319-41397-6_16

[72] Garland EL Jinpa T . Mindfulness-induced self-transcendence promotes universal love with consequent effects on opioid misuse. Behav Res Ther. (2024) 175:104494. doi: 10.1016/j.brat.2024.104494 38395015

[73] Vago DR Silbersweig DA . Self-awareness, self-regulation, and self- transcendence (S-ART): a framework for understanding the neurobiological mechanisms of mindfulness. Front Hum Neurosci. (2012) 6:296. doi: 10.3389/fnhum.2012.00296 23112770 PMC3480633

[74] Tang Y-Y Hölzel BK Posner MI . The neuroscience of mindfulness meditation. Nature Reviews Neuroscience. (2015) 16:213–225. doi: 10.1038/nrn3916 25783612

[75] Walser RD Evans WR Farnsworth JK Drescher KD . Initial steps in developing acceptance and commitment therapy for moral injury among combat veterans: Two pilot studies. J Context. Behav Sci. (2024) 32:100733. doi: 10.1016/j.jcbs.2024.100733 38826717

[76] Lathan EC Sheikh IS Guelfo A Choucair KC Fulton T Julian J . Moral injury appraisals and dissociation: Associations in a sample of trauma-exposed community members. J Trauma Dissoc.: Off J Int Soc For. Study. Dissoc. (ISSD). (2023) 24:692–711. doi: 10.1080/15299732.2023.2231010 37387238 PMC10771817

[77] Dambrun M . Self-centeredness and selflessness: Happiness correlates and mediating psychological processes. PeerJ. (2017). doi: 10.7717/peerj.3306 28507820 PMC5429736

[78] Brémault-Phillips S Cherwick T Smith-MacDonald LA Huh J Vermetten E . Forgiveness: A key component of healing from moral injury? Frontiers in Psychiatry. (2022) 13:906945. doi: 10.3389/fpsyt.2022.906945 35911220 PMC9328408

[79] Pernicano PU Wortmann J Haynes K . Acceptance and forgiveness therapy for veterans with moral injury: Spiritual and psychological collaboration in group treatment. J Health Care Chaplaincy. (2022) 28:S57–78. doi: 10.1080/08854726.2022.2032982 35135436

[80] Pizarro JJ Basabe N Fernández I Carrera P Apodaca P Man Ging CI . Self-transcendent emotions and their social effects: Awe, elevation and Kama Muta promote a human identification and motivations to help others. Front Psychol. (2021) 12:709859. doi: 10.3389/fpsyg.2021.709859 34589024 PMC8473748

[81] De Oliveira P Juneau C Stinus C Corman M Michelli N Pellerin N . Cultivating self-transcendence through meditation practice: A test of the role of meta-awareness, (Dis) identification and non-reactivity. psychol Rep. (2024). doi: 10.1177/00332941241246469 38669443

[82] Hanley AW Garland EL . Spatial frame of reference as a phenomenological feature of self-transcendence: Measurement and manipulation through mindfulness meditation. In: Psychology of consciousness: theory, research, and practice, vol. 6. (2019). p. 329. doi: 10.1037/cns0000204

[83] Molendijk T . Warnings against romanticising moral injury. British Journal of Psychiatry. (2022) 220:1–3. doi: 10.1192/bjp.2021.114 35045903

[84] Hall S . Comorbidities of combat trauma: Unresolved grief and moral injury. J Loss. Trauma. (2023) 28:51–60. doi: 10.1080/15325024.2022.2053227 37339054

[85] Kelley ML Bravo AJ Burgin EE Gaylord SA Vinci C Strowger M . Using mindfulness to manage moral injury in veterans: Feasibility and satisfaction of a pilot randomized controlled trial. J Clin Psychol. (2025) 81:425–33. doi: 10.1002/jclp.23778 40017139 PMC12050104

[86] Kabat-Zinn J . Full catastrophe living (rev. ed.): How to cope with stress, pain, and illness using mindfulness meditation. London, UK: Hachette UK (2013).

[87] Hayes SC Strosahl KD Wilson KG . Acceptance and commitment therapy: The process and practice of mindful change. New York, NY: The Guilford Press (2012).

[88] Twohig MP Vilardaga JCP Levin ME Hayes SC . Changes in psychological flexibility during acceptance and commitment therapy for obsessive compulsive disorder. J Context. Behav Sci. (2015) 4:196–201. doi: 10.1016/j.jcbs.2015.07.001 38826717

[89] Yadavaia JE Hayes SC Vilardaga R . Using acceptance and commitment therapy to increase self-compassion: a randomized controlled trial. J Context. Behav Sci. (2014) 3:248–57. doi: 10.1016/j.jcbs.2014.09.002 25506545 PMC4260406

[90] Litz BT Yeterian J Berke D Lang AJ Gray MJ Nienow T . A controlled trial of adaptive disclosure- enhanced to improve functioning and treat posttraumatic stress disorder. J Consult. Clin Psychol. (2024) 92:150–64. doi: 10.1037/ccp0000873 38358703 PMC11771448

[91] Gray MJ Schorr Y Nash W Lebowitz L Amidon A Lansing A . Adaptive disclosure: An open trial of a novel exposure-based intervention for service members with combat-related psychological stress injuries. Behav Ther. (2012) 43:407–15. doi: 10.1016/j.beth.2011.09.001 22440075

[92] Burkman K Gloria R Mehlman H Maguen S . Treatment for moral injury: impact of killing in war. Curr Treat Opt. Psychiatry. (2022) 9:101–14. doi: 10.1007/s40501-022-00262-6 35572857 PMC9078088

[93] Winkeljohn Black S Klinger K . Building spiritual strength: a spiritually integrated approach to treating moral injury. Curr Treat Opt. Psychiatry. (2022) 9:313–20. doi: 10.1007/s40501-022-00276-0 36466719 PMC9685023

[94] Harris JI Usset T Voecks C Thuras P Currier J Erbes C . Spiritually integrated care for PTSD: A randomized controlled trial of “Building Spiritual Strength. Psychiatry Res. (2018) 267:420–8. doi: 10.1016/j.psychres.2018.06.045 29980120

[95] Dahl CJ Lutz A Davidson RJ . Reconstructing and deconstructing the self: Cognitive mechanisms in meditation practice. Trends Cognit Sci. (2015) 19:515–23. doi: 10.1016/j.tics.2015.07.001 26231761 PMC4595910

[96] Johnson MW Richards WA Griffiths RR . Human hallucinogen research: Guidelines for safety. J Psychopharmacol. (2008) 22:603–20. doi: 10.1177/0269881108093587 18593734 PMC3056407

[97] Griffiths RR Richards WA McCann U Jesse R . Psilocybin can occasion mystical-type experiences having substantial and sustained personal meaning and spiritual significance. Psychopharmacology. (2006) 187:268–73. doi: 10.1007/s00213-006-0457-5 16826400

[98] Keltner D Sauter D Tracy JL Wetchler E Cowen AS . How emotions, relationships, and culture constitute each other: Advances in social functionalist theory. Cognition and Emotion. (2022) 36(3):388–401. doi: 10.1080/02699931.2022.2047009 35639090

[99] Watts R Day C Krzanowski J Nutt D Carhart-Harris R . Patients’ accounts of increased “connectedness” and “acceptance” after psilocybin for treatment-resistant depression. J Humanist. Psychol. (2017) 57:520–64. doi: 10.1177/0022167817709585

[100] Baer R Crane C Miller E Kuyken W . Doing no harm in mindfulness-based programs: Conceptual issues and empirical findings. Clin Psychol Rev. (2019) 71:101–14. doi: 10.1016/j.cpr.2019.01.001 30638824 PMC6575147

[101] Barkan T Hoerger M Gallegos AM Turiano NA Duberstein PR Moynihan JA . Personality predicts utilization of mindfulness-based stress reduction during and post-intervention in a community sample of older adults. J Altern Complement. Med. (2016) 22:390–5. doi: 10.1089/acm.2015.0177 27031734 PMC4860670

[102] Jiang T Sedikides C . Awe motivates authentic-self pursuit via self-transcendence: Implications for prosociality. J Pers Soc Psychol. (2022) 123:576–96. doi: 10.1037/pspi0000381 34855435

[103] Yaden DB Iwry J Slack KJ Eichstaedt JC Zhao Y Vaillant GE . The overview effect: awe and self-transcendent experience in space flight. Psychol Conscious.: Theory. Res. Pract. (2016) 3:1. doi: 10.1037/cns0000086 27371692

[104] Chirico A Ferrise F Cordella L Gaggioli A . Designing awe in virtual reality: an experimental study. Front Psychol. (2018) 8:2351. doi: 10.3389/fpsyg.2017.02351 29403409 PMC5786556

[105] Pérez KA Lench HC Thompson CG North S . Experimental elicitations of awe: A meta-analysis. Cogn Emotion. (2022) 37:18–33. doi: 10.1080/02699931.2022.2140126 36331080

[106] Johnson MW Garcia-Romeu A Cosimano MP Griffiths R . Pilot study of the 5-HT2AR agonist psilocybin in the treatment of tobacco addiction. J Psychopharmacol (Oxf Engl). (2014) 28:983–92. doi: 10.1177/0269881114548296 25213996 PMC4286320

[107] Breeksema JJ Niemeijer AR Krediet E Vermetten E Schoevers RA . Psychedelic treatments for psychiatric disorders: A systematic review and thematic synthesis of patient experiences in qualitative studies. CNS Drugs. (2020) 34:925–46. doi: 10.1007/s40263-020-00748-y 32803732 PMC7447679

[108] Morland L Woolley J . Psychedelic-assisted therapy for PTSD. National Center for PTSD, U.S. Department of Veterans Affairs. Washington, DC; (2025). https://www.ptsd.va.gov/professional/treat/txessentials/psychedelics_assisted_therapy.asp (Accessed September 15, 2025).

[109] Reed PG . Demystifying self-transcendence for mental health nursing practice and research. Arch Psychiatr Nurs. (2009) 23:397–400. doi: 10.1016/j.apnu.2009.06.006 19766931

[110] Goldberg SB Riordan KM Sun S Davidson RJ . The empirical status of mindfulness-based interventions: A systematic review of 44 meta-analyses of randomized controlled trials. Perspect psychol Sci. (2022) 17:108–30. doi: 10.1177/1745691620968771 33593124 PMC8364929

[111] Carey LB Hodgson TJ . Chaplaincy, spiritual care and moral injury: Considerations regarding screening and treatment. Front Psychiatry. (2018) 9:619. doi: 10.3389/fpsyt.2018.00619 30568605 PMC6290645

[112] Jones KA Freijah I Carey L Carleton RN Devenish-Meares P Dell L . Moral injury, chaplaincy and mental health provider approaches to treatment: A scoping review. J Relig. Health. (2022) 61:1051–94. doi: 10.1007/s10943-022-01534-4 35290554 PMC8922078

[113] Parameshwaran R . Theory and practice of chaplain's spiritual care process: A psychiatrist's experiences of chaplaincy and conceptualizing trans-personal model of mindfulness. Indian J Psychiatry. (2015) 57:21–9. doi: 10.4103/0019-5545.148511 25657453 PMC4314912

[114] Smigelsky, Melissa Trimm, Victoria Meador, Keith Jackson, George Wortmann, Jennifer Nieuwsma, Jason . Core components of moral injury groups co- facilitated by mental health providers and chaplains. Spiritual. Clin Pract. (2022) 9. doi: 10.1037/scp0000297 37360983 PMC10288643

[115] Aldwin CM Igarashi H Levenson MR . Wisdom as self-transcendence. In: Sternberg RJ Glück J , editors.The Cambridge handbook of wisdom. Cambridge, England, United Kingdom: Cambridge University Press (2019). p. 122–43. doi: 10.1017/9781108568272.007

[116] Lutz A Slagter HA Dunne JD Davidson RJ . Attention regulation and monitoring in meditation. Trends Cognit Sci. (2008) 12:163–9. doi: 10.1016/j.tics.2008.01.005 18329323 PMC2693206

[117] Kelley ML Strowger M Gabelmann JM Vasic S Rivera IE Fleming R . A review of empirical treatments focused on mind-body and spiritually grounded complementary practices for moral injury among veterans. Couns Val. (2024) 69:65–91. doi: 10.1163/2161007x-bja10011 PMC11502141 39450350

[118] Neimeyer RA . Meaning reconstruction in the wake of loss: Evolution of a research program. Behav Change. (2016) 33:65–79. doi: 10.1017/bec.2016.4 41292463

[119] Carlson EB Putnam FW . Dissociative experiences scale-II (DES-II) [Database record. APA. PsycTests. (1993). doi: 10.1037/t86316-000 27371692

[120] Kobayashi H Nemoto T Koshikawa H Osono Y Yamazawa R Murakami M . A self-reported instrument for prodromal symptoms of psychosis: Testing the clinical validity of the PRIME Screen-Revised (PS-R) in a Japanese population. Schizophr Res. (2008) 106:356–62. doi: 10.1016/j.schres.2008.08.018 18809299

[121] Brown KW Ryan RM . The benefits of being present: Mindfulness and its role in psychological well-being. J Pers Soc Psychol. (2003) 84:822. doi: 10.1037/0022-3514.84.4.822 12703651

